# Reactive arthritis developing after pneumococcal conjunctivitis: a case report

**DOI:** 10.1186/1752-1947-1-2

**Published:** 2007-02-02

**Authors:** Amit S Verma, Dorian Dwarika

**Affiliations:** 1 Western General Hospital, Crewe Road, Edinburgh, UK; 2 City Hospital, Dudley Road, Birmingham, UK

## Abstract

**Background:**

*S. pneumoniae *is a known cause of bacterial conjunctivitis and can be transmitted through contact with infected carriers.

**Case presentation:**

A 38-year-old ophthalmologist developing reactive arthritis following clinic-acquired pneumococcal conjunctivitis.

**Conclusion:**

(1) Despite the frequency and largely self-limiting nature of infective conjunctivitis, it should be appropriately assessed and managed, as the natural history can occasionally be associated with significant morbidity. (2) Hygienic measures are required to be implemented by both patients and ophthalmic staff to reduce the likelihood of transmission.

## Background

*Streptococcus pneumoniae *is a known cause of bacterial conjunctivitis. Outbreaks associated with this pathogen have been reported in the medical literature within the past few years [1-3]. Spread through contact with infected carriers has been implicated, as has contact lens wear [1].

We describe the case of an ophthalmologist developing the previously unreported complication of seronegative reactive arthritis after clinic-acquired bacterial conjunctivitis. This case highlights both the potential hazards faced by staff within the clinic setting and the possible morbidity associated with this condition.

## Case presentation

A 38 year-old male ophthalmologist was seen at the Ophthalmic Accident and Emergency Department with a five-hour history of a right watery red eye. He had been exposed to several conjunctivitis patients during the preceding few days, whilst working at the same department.

An initial diagnosis of probable viral conjunctivitis was made, and the patient was prescribed chloramphenicol eye drops. Conjunctival swabs were taken and sent to the microbiology laboratory for analysis. A day later the conjunctivitis had become bilateral and purulent. Several hours after the conjunctivitis became bilateral the patient also developed a sore throat. The previous swab cultures isolated *Streptococcus pneumoniae *as the infective pathogen. Three days later, the patient noticed tenderness and swelling of the proximal interphalangeal joint of the index finger in the left hand, which became progressively more swollen and tender over the next couple of days. The patient had become febrile; however, there were neither chills nor rigors. He was prescribed oral amoxicillin 500 mg TID and flucloxacillin 500 mg QID, in the belief this was septic arthritis.

Two days after the swelling started he was seen by a rheumatologist, who made a diagnosis of reactive arthritis. The patient was fully investigated (see following text and Table 1). Plain X-ray showed a little soft tissue swelling over the proximal interphalangeal joint of the left index finger. The patient was negative for antineutrophil cytoplasmic antibodies (ANCA), rheumatoid factor (RF), anti-DNA antibodies and antinuclear antibodies; complement factors C3 and C4 were within the reference range. No pathogen could be grown in blood culture and joint aspiration (performed by the rheumatologist) yielded no synovial fluid. The patient was HLA-B27 negative. A firm diagnosis of reactive arthritis as a sequel to pneumococcal conjunctivitis was made. Anti-inflammatory drugs resolved the swelling and pain within 48 hours. The conjunctivitis had already recovered though the antibiotics were continued until the course was completed.

**Table 1:** Selected blood test results. Values both at the time of initial investigation and after two months following the start of treatment with anti-inflammatory drugs.

Test	Blood test result at time of diagnosis	Blood test result after 2 months	Reference range (and units)
**White Cell Count**	4.7	4.4	3.7 – 9.5 (× 10^9^/litre)
**Neutrophils**	2.01	1.65	1.7 – 6.1 (× 10^9^/litre)
**Lymphocytes**	2.07	2.25	1 – 3.2 (× 10^9^/litre)
**Monocytes**	0.25	0.21	0.2 – 0.6 (× 10^9^/litre)
**Eosinophils**	0.25	0.15	0.03–0.46(× 10^9^/litre)
**Basophils**	0.02	0.03	0.02–0.09 (× 10^9^/litre)
**Platelets**	308	269	143 – 400 (× 10^9^/litre)
**CRP**	< 5	< 5	0 – 5 (mg/litre)
**ESR**	17	8	1 – 10 (mm/hour)

## Discussion and conclusion

Reactive arthritis was initially used to describe the development of sterile inflammatory arthritis as a sequel to remote infection. Although no specific criteria exist for the diagnosis of reactive arthritis, it is often based clinically on the development of acute oligoarticular arthritis within 2–4 weeks of a preceding infection. [4]. Over the past two decades, a clinical entity termed 'post-streptococcal reactive arthritis' (PSRA) has been used to describe a cluster of symptoms sharing features with both acute rheumatic fever and HLA-B27-related spondyloarthropathies. Although sore throat is the common feature and the most frequent site for positive cultures is the throat, streptococcal isolates have been also been obtained from blood cultures and high vaginal/endocervical swabs [5]. Whilst conjunctivitis has been reported as an associated feature in three PSRA cases [6,7], we believe this is the first report of reactive arthritis developing as a consequence of primary pneumococcal conjunctivitis.

Reactive arthritis has usually been described in the context of preceding urogenital or enteric infections. However, in common with both urogenital and enteric infections, streptococci can invade mucosal surfaces. Cell surface components and extracellular products facilitate streptococcal tissue invasion [8]. The precise mechanism(s) by which reactive arthritis could develop remain uncertain [9].

Hygienic measures such as frequent hand washing, disinfection of ophthalmic equipment particularly after infected cases, and the use of disposable tonometer prisms are designed to reduce the risk of infection transmission in the clinic setting. Studies have shown that hand hygiene is not always practised by a substantial proportion of ophthalmic healthcare workers [10] and that ophthalmologists can carry a significant microbial load on their hands [11]. This case shows the risk faced by healthcare workers whilst treating infected patients and highlights the importance of the implementation of hygienic measures by both patients (through education on the importance of frequent hand washing and avoiding the sharing of eating utensils/towels) and healthcare workers to reduce the risk of transmission. It also emphasizes that regardless of the frequency of presentation; rare serious consequences of infective conjunctivitis can and do occur and need to be recognised and managed appropriately, using a multi-disciplinary approach if required.

## Competing interests

The author(s) declare that they have no competing interests.

## Authors' contributions

All authors were involved in writing/reviewing the manuscript. All authors approved the final manuscript.

## References

[B1] Martin M Turco JH Zegans ME Facklam RR Sodha S Elliot JA Pryor JH Beall B Erdman DD Baumgartner YY Sanchez PA Schwatrzman JD Montero J Schuchat A Whitney CG An Outbreak of Conjunctivitis Due to Atypical Streptococcus pneumoniae N Engl J Med 2003 348 1112 1121 10.1056/NEJMoa022521 12646668

[B2] Centers for Disease Control and Prevention Outbreak of Bacterial Conjunctivitis at a College-New Hampshire, January-March, 2002 Morb Mortal Wkly Rep 2002 51 205 207 11922188

[B3] Centers for Disease Control and Prevention Pneumococcal Conjunctivitis at an Elementary School – Maine, September 20-December 6, 2002 Morb Mortal Wkly Rep 2003 52 64 66 12578323

[B4] Hannu T Inman R Granfors K Leirisalo-Repo L Reactive arthritis or post-infectious arthritis? Best Pract Res Clin Rheumatol 2006 20 419 33 10.1016/j.berh.2006.02.003 16777574

[B5] Mackie SL Keat A Poststreptococcal reactive arthritis: what is it and how do we know? Rheumatology 2004 43 949 954 10.1093/rheumatology/keh225 15150434

[B6] Rogerson SJ Beeching NJ Reactive arthritis complicating group G streptococcal septicaemia J Infect 1990 20 155 158 10.1016/0163-4453(90)93470-D 2319147

[B7] Madhuri V Mathai E Brahmadathan KN Korula RJ John TJ An outbreak of post-streptococcal reactive arthritis Indian J Med Res 1997 105 249 253 9277035

[B8] Kadioglu A Andrew PW The innate immune response to pneumococcal lung infection: the untold story Trends Immunol 2004 25 143 149 10.1016/j.it.2003.12.006 15036042

[B9] Kim T-H Uhm W-S Inman RD Pathogenesis of ankylosing spondylitis and reactive arthritis Curr Opin Rheumatol 2005 17 400 405 10.1097/01.bor.0000163447.44037.c4 15956835

[B10] Mensah E Murdoch IE Binstead K Rotheram C Franks W Hand hygiene in routine glaucoma clinics Br J Ophthalmol 2005 89 1541 1542 10.1136/bjo.2005.072538 16234477 PMC1772957

[B11] Lam RF Hui M Leung DYL Chow VCY Lam BNM Leung GM Lam DSC Extent and Predictors of Microbial Hand Contamination in a Tertiary Care Ophthalmic Outpatient Practice Invest Ophthalmol Vis Sci 2005 46 3578 3583 10.1167/iovs.05-0216 16186336

